# Uncovering Evidence for Endocrine-Disrupting Chemicals That Elicit Differential Susceptibility through Gene-Environment Interactions

**DOI:** 10.3390/toxics9040077

**Published:** 2021-04-06

**Authors:** Dylan J. Wallis, Lisa Truong, Jane La Du, Robyn L. Tanguay, David M. Reif

**Affiliations:** 1 Department of Biological Sciences, North Carolina State University, Raleigh, NC 27695, USA; djwallis@ncsu.edu; 2 Department of Environmental and Molecular Toxicology, Oregon State University, Corvallis, OR 97331, USA; lisa.truong@oregonstate.edu (L.T.); jane.ladu@oregonstate.edu (J.L.D.); robyn.tanguay@oregonstate.edu (R.L.T.)

**Keywords:** endocrine-disrupting chemical (EDC), gene-environment interaction (GxE), differential susceptibility

## Abstract

Exposure to endocrine-disrupting chemicals (EDCs) is linked to myriad disorders, characterized by the disruption of the complex endocrine signaling pathways that govern development, physiology, and even behavior across the entire body. The mechanisms of endocrine disruption involve a complex system of pathways that communicate across the body to stimulate specific receptors that bind DNA and regulate the expression of a suite of genes. These mechanisms, including gene regulation, DNA binding, and protein binding, can be tied to differences in individual susceptibility across a genetically diverse population. In this review, we posit that EDCs causing such differential responses may be identified by looking for a signal of population variability after exposure. We begin by summarizing how the biology of EDCs has implications for genetically diverse populations. We then describe how gene-environment interactions (GxE) across the complex pathways of endocrine signaling could lead to differences in susceptibility. We survey examples in the literature of individual susceptibility differences to EDCs, pointing to a need for research in this area, especially regarding the exceedingly complex thyroid pathway. Following a discussion of experimental designs to better identify and study GxE across EDCs, we present a case study of a high-throughput screening signal of putative GxE within known endocrine disruptors. We conclude with a call for further, deeper analysis of the EDCs, particularly the thyroid disruptors, to identify if these chemicals participate in GxE leading to differences in susceptibility.

## 1. Introduction

The EDCs are a broad class of compounds that have emerged in the last several decades as an imminent threat to human health. Over ten years ago the Endocrine Society released their first Scientific Statement on EDCs [1]. Since the publication of that article, the number of papers published on these chemicals has increased every year. According to one study, the estimated cost of exposure to EDCs in the US was $340 billion dollars [2].

The Endocrine Society, in their second Scientific Statement, defines an endocrine-disrupting chemical as “*an exogenous chemical, or mixture of chemicals, that interferes with any aspect of hormone action*” [3]. This interference has been shown to cause a range of negative effects, including impacts on cardiovascular health, metabolic health, reproductive health, prostate gland function, thyroid function, and neurodevelopment [3,4,5,6]. Despite their deleterious effects, endocrine-disrupting chemicals are present in a wide array of consumer products that most people encounter every day, including cosmetics, personal care products, pesticides, packaging, and building materials [7,8,9,10,11,12,13].

Many diseases that are associated with EDCs, such as obesity and type 2 diabetes, have known genetic risk factors [14,15]. EDCs have diverse mechanisms of action. They can affect protein and gene expression via interaction with a multitude of biological pathways [3], suggesting the possibility of genetic differences being able to modulate the effects of endocrine disruptors via GxE.

Some of the effects of EDCs already have well-characterized gene-environment interactions that modulate an organism’s susceptibility [16,17,18,19]. This is only a drop in the bucket of the many endocrine-disrupting chemicals that may participate in GxE. The advent of toxicogenomics and personalized medicine underscores the importance of understanding individual differences across populations in the effects of chemical exposure [20,21]. Understanding which EDCs are involved in GxE and what genetic factors contribute to susceptibility can yield a better understanding of risk, a better grasp on the chronic and acute effects of a chemical, a more diverse regulatory environment, as well as increasing knowledge about the mechanisms by which a chemical disrupts biological pathways. This is especially important in endocrine disruptors because their complex and varied mechanisms and effects can often cause challenges to current research and risk assessment [22].

In this paper we will discuss endocrine-disrupting chemicals and the mechanisms by which they may cause differential susceptibility in a population that is caused by population-wide genetic differences, focusing on thyroid disruption. We will then discuss methods by which chemicals that display gene-based, population-wide differences can be identified, and then the genetic etiology of these differences can be found.

## 2. Biology of Endocrine-Disrupting Chemicals and Implications for Diverse Populations

### 2.1. History and Exposure

When diethylstilbestrol (DES) was found to cause cancer in the daughters of mothers that took it, a new era in toxicology had begun [23]. EDCs are now ubiquitous in the built environment [7,8,9,10,11,12,13]. They are present in a wide array of the products we use daily in many different forms that can affect the endocrine system in myriad ways. The most famous and first studied class of endocrine disruptors is estrogenic EDCs like DES [23,24].

### 2.2. Molecular Biology of EDCs

The study of EDCs has since gone beyond estrogen-active chemicals. Compounds have been identified that affect several of the nuclear hormone receptors (NR) including the estrogen receptor (ER), androgen receptor (AR), the glucocorticoid receptor (GR), the mineralocorticoid receptor (MR), the thyroid receptor (TR), and the peroxisome proliferator-activated receptor (PPAR) [25,26,27,28,29]. These receptors contain a ligand-binding domain (LBD), that recognizes and binds discrete ligands, and a DNA binding domain (DBD), which binds a specific DNA sequence as either a homodimer or heterodimer, after a ligand is bound [30]. The specific DNA sequence bound by an NR is known as a hormone response element (HRE) [30].

HREs, in general, are arranged as hexameric repeats [31]. These repeats can be direct, inverted, or everted hexamers with a spacer of length 3 or more in between them [31,32,33,34,35,36]. Within the nuclear receptors, these consensus sequences are often shared [33,37,38]. For example, the AR and GR share the exact same sequence as their response element [37,38]. Specificity of NR binding is imparted via a number of mechanisms. Experiments that created mutations in a given HRE show that changes in the consensus sequence, especially of the first hexamer in the repeat, can make an HRE more or less specific to a certain NR [33,36,39]. The distance between repeats can also change the specificity of an HRE [32,36]. The orientation or composition of an NR dimer can also change based on the orientation of the HRE [32,36]. Going beyond the consensus sequence of an HRE, the base pairs that flank the hexameric repeats may exert a level of specificity to an HRE [34].

Binding to their respective response elements allows NRs to modulate many physiological functions via transcriptional control of certain genes [40]. Endocrine-disrupting chemicals that act on NR can act as agonists, activating the receptor by mimicking its ligand, or as antagonists, in some way preventing the activity of the receptor [29,41,42,43]. Some well-known EDCs include bisphenol A, a widely discontinued plastic and resin additive; DDT, a pesticide that is used around the world; estradiol, the first endogenous estrogenic hormone to be synthesized; Genistein, a phytoestrogen; tamoxifen, a non-steroidal estrogen that has been used for breast cancer treatment; and phthalates, a class of chemicals used in many industrial applications [10,41,44,45,46,47]. As more chemicals are tested for endocrine disruption the list continues to grow. A recent effort by the EPA to screen almost 2000 chemicals using computational methods and comparing them to known estrogens identified 111 compounds for further investigation [43]. A similar study found 220 chemicals that exhibited AR receptor agonism or antagonism as well as 174 more chemicals that may be weakly active in the AR pathway [42]. While direct interactions with NRs and their binding sites are common mechanisms of endocrine disruption there are several other ways they may interact across these pathways that could be subject to genetic effects. These mechanisms include inhibition or stimulation of hormone synthesis, changes in the hormone binding, binding of hormone-binding proteins, and stimulation or inhibition of hormone-binding protein synthesis [47].

### 2.3. Effects on Biological Pathways

The many different EDCs, with their disparate functions and structures, can work via several different pathways based on which receptors they interact with. Estrogenic compounds, in some way, mimic endogenous compounds that bind to the ER. Depending on structure these chemicals can bind to either estrogen receptor alpha (ERα) or estrogen receptor beta (ERβ) causing differing effects [45,48]. Estrogen signaling affects several pathways and differs based on the localization of the ER in different tissues and locations within the cell [24,49]. Many estrogenic EDCs act as estrogen receptor agonists or antagonists throughout the body [24,45,48]. Some act differently depending on the tissue in which they are localized. These are known as selective estrogen receptor modulators or SERMs [44,50]. ERα and ERβ signaling has profound effects on multiple biological systems. Activation and antagonism of these receptors can affect the development and upkeep of sexual organs, the lungs, adipose tissue, the immune system, and the brain [51,52]. These effects are produced when these signals are propagated across a diverse array of signaling pathways [53].

The AR shares a superfamily designation with many other NRs including the ER [30]. Much like the ER, the AR plays an important role in the regulation and upkeep of important biological processes [54]. These processes include the development and function of sexual organs, cognitive and sexual behavior, brain patterning, hair patterning, and muscular development and upkeep [54,55,56,57,58,59]. The primary hormones that act as AR agonists are testosterone and 5α-dihydrotestosterone, a metabolite of testosterone [55]. Although anti-androgens are better studied, androgen disrupting chemicals can bind the AR and act as agonists or antagonists [26,42,60]. In addition to being modulated by ligands and having specific elements with high affinity, the AR is similar enough to the progestin and glucocorticoid receptor which allows to share response elements with them [54,61,62,63]. The AR can also bind a variety of response elements on the DNA via the differing orientation of the homodimer formed after ligand binding to modulate a number of genes [63].

The activity of thyroid hormones is regulated by a complex web of feedback loops. In short, the thyroid gland is stimulated to produce thyroid hormones via thyroid-stimulating hormone (TSH) from the pituitary [64,65]. Thyroid hormone receptors (TRs) are acted on by thyroid hormones around the body, primarily triiodothyronine (T3) and thyroxine (T4) [64]. TSH secretion is, itself, regulated by levels of thyroid hormone circulating in the bloodstream [64,65]. Thyroid hormone receptors (TRs) localize in multiple cell types across the body [65,66]. Much like the ER, the TR has multiple isoforms [65,66,67,68]. These isoforms are splice products that are coded by two different genes at two different chromosomes each one localizing and functioning in different ways [65,66,67,68]. TRα1, TRβ1, TRβ2, and TRβ3 all bind T3 while TRα2, TRα3 do not [67,68]. Beyond localization and structure, the effects of the TR can be further modulated by the recruitment of corepressors and coactivators, and interaction with other signaling pathways beyond the thyroid [69,70,71]. This complex system plays an important role in growth and development, neural differentiation, and metabolic regulation [64,65,71,72,73,74].

With such diverse pathways within which these nuclear hormones play a definitive role, endocrine disruptors could mediate a multitude of effects on biological systems, leading to substantial consequences to exposure. In fact, in the time that endocrine disruptors have been studied it has been shown that they may lead to metabolic, neurological, reproductive, and cardiovascular dysfunctions, as well as cancer [3]. Studies have shown that exposure to EDCs such as persistent organic pollutants (POPs) and DES, especially during development, can lead to obesity via interaction with nuclear hormone receptors [4,75]. Obesity itself can also lead to differences in the effects of EDC exposure. Endocrine disruptors are extremely lipophilic and are therefore stored in fat cells, this can lead to an increased body burden of endocrine disruptors in people who are obese [4]. Although limited, studies have tied autism spectrum disorder to prenatal exposure to EDCs [76]. Dichlorodiphenyldichloroethylene (*p*,*p*′-DDE) has been linked to an increased risk of diabetes [77,78]. EDCs have been shown to negatively affect neurological development after pre- and peri-natal exposure, oftentimes through disruption of the thyroid signaling pathway [79,80,81]. In fact, the life stage in which exposure occurs can drastically affect an individual’s susceptibility to that chemical [4,75,82,83,84]. If exposure occurs during a “window of susceptibility” or not can exacerbate or ameliorate the future effects of exposure [4,75,82,83,84]. The time in which someone is exposed to certain EDCs has been shown to have a large effect on the development of metabolic disorders such as obesity and type 1 diabetes, or if they develop specific cancers such as breast cancers [4,75,82,83,84]. These windows of susceptibility have a serious impact on how research into individual susceptibility to EDCs should be done. Life stages for research should be chosen carefully to capture the desired effect. Endocrine disruptors themselves, especially phthalates, have repeatedly been tied to childhood asthma [82,83,84]. BPA has been associated with reproductive dysfunction in humans [85]. Several studies tied dioxin to cardiovascular disease mortality [86]. As far as cancer is concerned, EDCs have been tied to the development of multiple types of cancer including vaginal, prostate, breast, ovarian, and cervical cancers [23,87,88,89,90,91,92,93]. A more detailed look into estrogenic, androgenic, and thyroid disruptors and their effects can be found in Kiyama and Wada-Kiyama [24], Luccio-Camelo and Prins [26], and Calsolaro et al. [94] and *The Endocrine Society’s Second Scientific Statement on Endocrine-Disrupting Chemicals* [3].

### 2.4. Implications for Population Diversity

With so many processes being regulated by the endocrine system and NRs themselves, it comes as no surprise that disruption of any of these systems can cause such a wide range of effects [50,51,52,53,54,55,56,57,58,63,64,65,66,67,68,69,70,71,72,73]. Not surprisingly, the effects of EDCs are not limited to any area of the body or a family of diseases [3,4,23,74,75,76,77,78,79,80,81,82,83,84,85,86,87,88,89]. The NRs exist within complex systems of tightly controlled interactions that modulate a diverse array of systems across the body [50,51,52,53,54,55,56,57,58,63,64,65,66,67,68,69,70,71,72,73]. These systems hinge on the interactions between the receptors and a small number of consensus sequences throughout the human genome [25,26,27,28,29,30,31,32,33,34,35,36,37,38,39,40,41,42,43,44,45,46]. These sequences, along with the sequences that code for the receptors, must be subject to the same standing variation in the population that exists across the human genome. These effects cascade across multiple biological systems that are also subject to the effects of changing genetics [50,51,52,53,54,55,56,57,58,63,64,65,66,67,68,69,70,71,72,73]. Differences at genetic loci in important genes have, repeatedly, been shown to be capable of causing population-level differences in susceptibility to diseases. Therefore, differences in the genetic loci that code for NRs or sequence differences in the HREs that they bind have the capability to lead to differences in susceptibility to EDCs across a genetically diverse population by affecting responses to EDC exposure.

## 3. Evidence for Differential Susceptibility to Endocrine-Disrupting Chemicals

The genetic nature of the NRs influence on complex biological systems implies the possibility of genetic differences having far-reaching effects on the influence of EDCs on different aspects of those same systems. While sparse, emerging studies suggest that genetic differences can lead to response differences following EDC exposures.

Several studies in rats have shown that differences across strains demonstrated a marked difference in perturbation to the endocrine system [95,96,97,98,99,100]. One rat study found that exposure to 2,4,5,2′,4′,5′-hexachlorobiphenyl (HCB) caused increased expression of multiple cytochrome p450 enzymes, but this response was suppressed by an endocrine mediated mechanism that was based partly on genetic differences in rat strains [97,98]. The endocrine-disrupting effects of 2,3,7,8-tetrachlorodibenzo-*p*-dioxin (TCDD) have also been linked to strain differences in rats, specifically relating to the aryl hydrocarbon receptor [99,100]. Strain differences in susceptibility to estrogen disruption in mice, specifically relating to breast cancer, have been studied and shown extensively across various strains of mice [17,18,101,102]. Many studies have also examined gene-environment interactions as they relate to human reproductive function. Many different chemicals have been shown to cause variable phenotypes within effects like cryptorchidism, hypospadias, testicular dysgenesis, and infertility [16,103]. Yet even these may need further investigation [16].

Compared to many biological systems, the thyroid signaling process is extremely complicated: it affects almost all cells in vertebrates, it’s regulated by a complex system of feedback loops, and it contains multiple isoforms and ligands that control localization and effects of TR binding molecules [64,65,66,67,68,69,70,71,72,73,74]. As a result, in many cases, differential susceptibility to the thyroid effects of endocrine disruptors tends to be overlooked. Yet evidence of a genetic basis for differential susceptibility to many exposures via the thyroid system does exist within the literature. A study on asthma patients found a SNP 5′ to the thyroid receptor beta gene that was associated with the patient response to isopterenol [104]. One small study indicated that polymorphisms in the THRA and THRB genes were associated with autism spectrum disorder [105]. Together this may represent an indication that there are genes that create susceptibility to prenatal thyroid disruption leading to an adverse outcome. Some studies have pointed to genetic variants that may cause changes in sensitivity to thyroid hormone [106,107]. Mutations upstream of the THRB gene are associated with resistance to thyroid signaling [106,107]. One gene variant on the promoter region of a tumor necrosis factor gene, considered a risk allele for joint disorders, acts as a thyroid receptor binding site [108]. One variant at this locus appears to sensitize the region to certain stimuli, leading to joint problems [108]. This is a clear example of increased susceptibility due to a genetic variant within the thyroid signaling system. Studies in rats have also indicated clear strain differences in susceptibility to thyroid disruption. One study showed that different strains of rats showed differences in individual susceptibility to developing thyroiditis after irradiation and thymectomy [96]. Another study found that genetic differences in rats led to marked differences in responses to iodine deficiency [95]. Mutations in the thyroid receptor genes have also been tied to various varieties of cancer [109,110,111]. Differential responses within the thyroid signaling system are even of importance to agriculture. Differences in response to T_3_ supplementation have been shown in various strains of chickens [112]. One promising study looking at susceptibility to T_3_ used molecular modeling combined with site-directed mutagenesis to investigate mutations in the thyroid hormone receptors that could influence T_3_ binding [113]. This study is a step in the right direction towards understanding differences in susceptibility to thyroid disruption.

**Figure 1:**
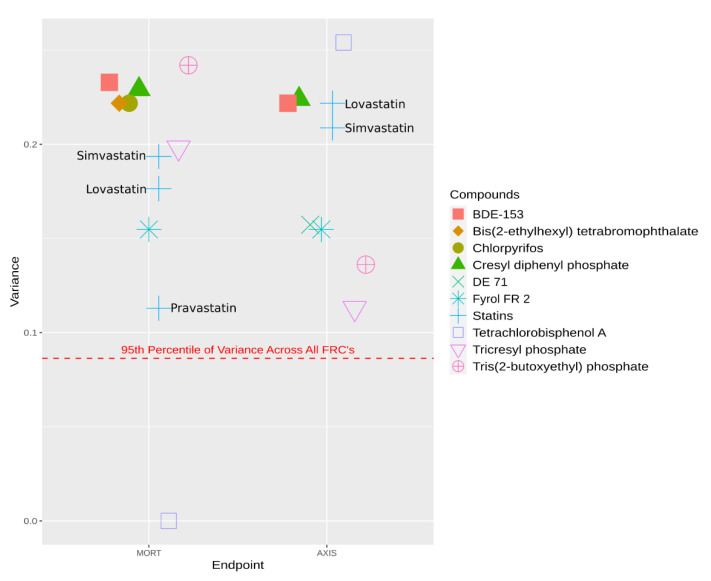
Variance across a high-throughput screening assay in a genetically diverse population of zebrafish across two endpoints: mortality (MORT) and bent axis (AXIS) at a chemicals lowest effect level as measured in the screening. This includes flame retardant chemicals (FRCs) that are enriched in thyroid pathways as well as statins, which are known to participate in GxE which lead to differences in susceptibility, for comparison. The statins are individually labelled. The line on the bottom represents the 95th percentile of variance in the full FRC screening. Each chemical is a different shape and color.

A class of chemicals that have been widely identified as endocrine, and specifically thyroid, disruptors is the flame retardant chemicals (FRCs) [114,115,116,117]. One study screened flame retardants for developmental effects on a genetically diverse zebrafish population [118]. High variance within an endpoint across a genetically diverse population could represent GxE and be driven, partially, by heritable traits that make the fish more or less resistant to exposure. Reif et al. showed that by identifying chemicals in a high throughput screening that showed high variance in multiple endpoints, one could find chemicals that exhibited GxE that modulates susceptibility [119]. Closer examination of the data from the flame-retardant screening study shows that many of these flame retardants have a high variance of effect across the genetically diverse fish population. This is represented in Figure 1 which shows multiple high variances across two prevalent endpoints in FRCs at their measured lowest effect level (LEL) compared to the variability across all FRCs. Figure 1 also includes the variance of statins across a high-throughput zebrafish screening [118,119]. Statins have been shown to participate in GxE affecting the individual susceptibility to statins across the human population via transport pathways [120,121]. Statins also exhibit high variance across the same genetically diverse zebrafish population, according to another high-throughput screening study. Statins are plotted with the FRCs in Figure 1 to illustrate that the variance of effect across the population within FRCs is similar to a chemical with known GxE. 

**Figure 2:**
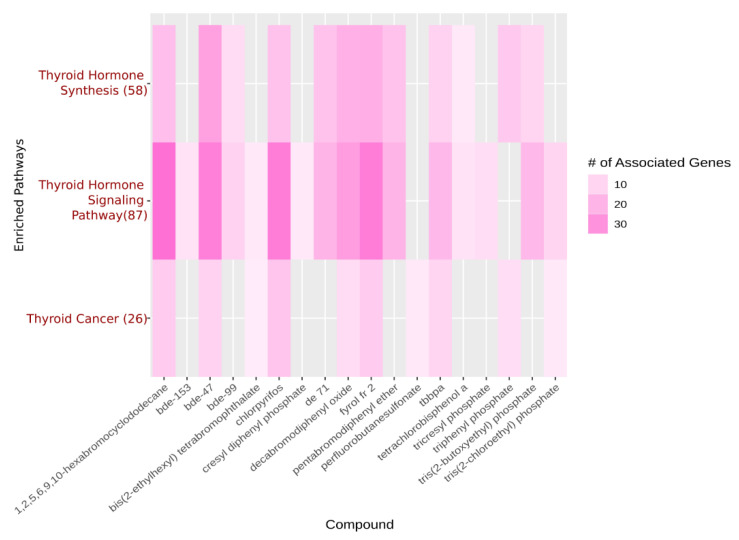
A heatmap displaying a pathway enrichment analysis done on CTD using FRCs that were used in a high throughput screening experiment. Individual FRCs are plotted along the *x*-axis. The *y*-axis lists the pathways that are enriched for these chemicals, with the number in parentheses indicating the number of genes in each pathway that these chemicals (taken as a whole) are enriched in. Darker coloration represents increasing numbers of genes that are associated with each FRC.

The Comparative Toxicogenomics Database (CTD) shows that statins are enriched across multiple transport pathways in humans [122]. FRCs are enriched, as a whole, in several thyroid pathways, and several of the screened FRCs are shown to be individually enriched in these same thyroid pathways [118,122]. This is illustrated in Figure 2. 

This combined enrichment, as a class and individually, in addition to high variance across a genetically diverse population may represent a signature of GxE in a chemical class. In addition, many of the highest variance flame retardant chemicals identified by this simple analysis (Figure 2), already have several studies showing endocrine-disrupting effects [123,124,125,126,127,128,129,130,131,132,133,134,135,136,137,138,139,140,141,142,143,144,145,146,147,148,149,150,151,152]. Looking at the variance of an endpoint in a high throughput screening experiment represents one way to quickly identify candidate chemicals for GxE. The FRCs represent a class of endocrine disruptors that affect the thyroid and exhibit a signature of effects that could be representative of EDCs that participate in GxE and lead to differential individual susceptibility across a population. It certainly warrants further investigation as to what classes of EDCs should be considered for deeper analysis into the possibility that they can cause a wide range of effects based on small genetic differences.

## 4. Characterizing GxE Effects Associated with Endocrine-Disrupting Chemicals

All of this information points to the strong possibility that gene-environment interactions may play a role in susceptibility to endocrine disruption by environmental exposures, but what is GxE? GxE is, simply put, how environmental exposures can be modulated by interaction with specific genes. The result being that one individual may respond differently to some environmental exposure than another individual in the same population with a different allele or set of alleles. As we have seen, the endocrine system is highly subject to the influence of genes in normal function as well as an individual’s response to exposure. Along with other factors, GxE is implicated as an important contributor to multiple complex diseases including asthma, cardiovascular disease, developmental defects, diabetes, autism spectrum disorder, reproductive defects, and cancer [16,104,153,154,155,156,157,158,159,160,161]. Many of these diseases have also been tied to endocrine disruptors [76,78,79,84,85,87,89,90,91,92,94,99,100]. Studying how gene-environment interactions influence the effects of endocrine disruption could lead to breakthroughs in prevention and treatment of exposure as well as an expanded knowledge base on the mechanisms of these effects.

The interest in gene-environment interactions has been steadily growing, partially due to the advent of affordable and fast next-generation sequencing techniques and the methods to analyze them [157]. GxE studies have proven to be challenging. Oftentimes these studies require large sample sizes and the exposures can be hard to validate or pin down when using human data [162]. Over time though, many effective approaches have been developed to identify GxE. Many GxE study designs have been adopted in the search for GxE. These studies can use data from consortia or single studies and are most often genome-wide association studies using large sample size and can include case-control, case-only, and family data frameworks [119,163,164,165,166,167,168].

These studies require complex analytical methods to extract information on interactions between the proposed exposures and possible interactions. In recent years, many exciting, and effective methods have begun to arise that increase the power of investigations into GxE.

The earliest experimental designs in genetic studies used family data to try and identify genetic etiologies of disease [169]. This study design has also been leveraged to identify gene-environment interactions [169]. Family data is still important to the field and is used to this day, often in conjunction with sequencing data, to find GxE [166,167]. One study evaluated three different approaches to data analysis, implementing generalized estimating equations (GEE), a hierarchical linear model, and a pedigree-based mixed modeling approach in R, three popular statistical approaches in GxE analysis [166]. This study concluded that these approaches are all comparable in their results but showed that the GEE model may have had increased type-I error [166]. Another study also analyzed the GEE model for family data along with linear mixed-effects models, finding that GEE is more robust against misspecification of exposure, a risk in GxE studies [167]. With the advent of more complex genetic data, the preferred design of GxE studies became the case-control design using unrelated individuals [164]. This design utilizes exposure that causes a specific phenotype and compares individuals who display that phenotype to unaffected individuals.

Wu et al. (2018) attempted to deal with the often hierarchical “main effects, interactions” structure that exists in many GxEs [170]. The researchers assessed the effectiveness of an approach that uses a penalized method with the least absolute deviation loss function, using a simulation and a case study and found that the approach performed well in these circumstances [170]. To overcome the challenges that exist when trying to find rare variants with binary phenotypes, one study used a similarity-based regression method for evaluating GxE interactions that combined data using genetic similarity across markers [129]. Beyond misclassification of exposure, another issue that can arise is the complexity of dealing with multiple exposures. One study proposes a linear mixed model to overcome this [171]. Oftentimes permutation tests are done to avoid error, although one such study used a parametric bootstrap model to avoid the need for permutation tests when that was not possible [164,172]. One emerging approach to try and find significant conclusions from such studies is meta-analysis [173]. Meta-analysis-based approaches can leverage the results from multiple similar studies to try and find GxE [173].

One early paper exploring GxE methods that used linear modeling and logistic regression for analysis suggests that in the specific scenario that the phenotype is rare, and the causative exposure is independent of the genetics, removing individuals without the phenotype of interest could increase precision [174]. This case-only analysis method has evolved over the years into a tool that can be used to effectively find gene-environment interactions. One problem that arises in case-only analysis is the independence assumption [175,176,177]. Significant bias can be introduced when the independence of the genetics and the exposure are misclassified [168,175,177]. Soon after this initial study Umbach and Weinberg [175] and Chatterjee and Carroll [178] expanded on the method by using multiple maximum likelihood methods for case-only analysis. These studies included scenarios where only cases were sampled, and situations where the independence of exposure and genetics is conditional allowing for some relaxation of the independence assumption. Mukherjee and Chatterjee took advantage of Bayesian methods to relax the independence assumption while maintaining the increased precision of the case-only method [177].

Motivated by the improvements demonstrated by the case-only method while understanding that the independence assumption could, sometimes, not be applied, Murcray et al. proposed a 2-step method that utilized the methods developed for case-only analysis [176]. The first step uses a likelihood ratio test of association between G and E, taken from case-only tests, to screen for initial SNPs in GWAS data and then uses a traditional GxE test on the SNPs that pass in step 1 [176]. This method utilizes a decrease in the usual multiple testing correction for increased power [176]. This is true even in the case of some G-E dependence because the second step is necessarily unbiased even if the first step incurs some bias [176]. Paré et al. introduced a 2-step method that screens by differences in variance between traits in different genotypes using Levene’s test [179]. Zhang et al. compared this method to a method from Kooperburg and LeBlanc, which proposes a 2-step method that leverages marginal effects to screen for gene × gene interactions, in order to find an effective 2-step method to discover GxE [165,180]. They then introduce two new methods: a modified version of the Paré et al. approach looking at variance heterogeneity in the residuals rather than the trait, and a combined version of the two original methods [165]. While all of these approaches provide a robust approach to finding GxE, the evaluation found that in certain situations the Paré et al. approach can have increased type I error [165]. In the case where more than one interaction occurs the variance heterogeneity approach and the joint approach showed increased power over the other methods but were similar to the other approaches or less powerful when only one interaction occurred [165].

Biernacka et al. expanded the 2-step method further by introducing the use of gene-set analysis (GSA) in tandem with a primary screening step [181]. This gene-set approach was initially used for gene-expression studies but is emerging as a powerful method to analyze GWAS data [182,183,184,185]. Single-nucleotide polymorphism (SNP) approaches to GWAS are often limited by sample size and may not have the power to find rare variants or variants with small effects [183,186]. Gene-sets can be constructed out of SNPs that are associated based on linkage or biological pathways [183]. GSA can be considered self-contained, wherein only the gene-sets are analyzed, and competitive, where SNPs are first analyzed by themselves for significance [182,183,186]. Fridley and Biernacka provide an in-depth review of all of the gene-set methods used for GWAS, including one- and two-step methods, introduced prior to the paper’s publishing [182]. One study found that when compared, two-step analysis with gene-sets were more powerful than one-step methods [181]. These GSA approaches have been parlayed into a useful approach to identify GxE [184,185]. Tzeng et al. proposed a method to combine SNPs via similarity regression to build a marker set that can be used to identify GxE [187]. This method allows for the investigation of rare and common variants, can account for multiple covariates, and prevents signals that are in opposite directions cancelling each other out [182]. Lin et al. proposed a gene-environment set association test (GESAT) that uses a variance component test to find SNP-set environment interactions along with ridge regression to identify main effects [185]. An easy-to-use tool, MAGMA, has even been expanded to do gene-set analysis for GxE [184].

The problems that plague the search for GxE, including small sample sizes, underpowered methods, and misunderstood interaction can contribute to erroneous developments and missed conclusions. To identify GxE that affects susceptibility to endocrine disruptors, the methods used should be well understood and the approach should be tailored to each problem. In the case of susceptibility to thyroid disruption, an under-investigated problem, the issue is many-faceted. The thyroid system is made up of and connected to many complex pathways, the thyroid receptor itself has several isoforms that are known to behave slightly differently. This lends itself to a gene-set analysis method that combines SNPs into pathways, combined with a 2-step approach would allow for a robust and powerful approach to investigating susceptibility to thyroid disruption. Ritchie et al. reviewed methods to include biological knowledge, improving the effectiveness of GxE studies, using approaches that include databases, endophenotypes, and model organisms [188]. The methods discussed in this paper could be used to simplify the complexity of the thyroid pathways to better find GxE that contributes to susceptibility. Searching the thyroid signaling pathway alone the CTD brings up 469 diseases tied to a great many more chemicals, some known endocrine disruptors, but a comparatively smaller 116 genes [122]. Using databases like this could help simplify the analysis of GxE, increasing power these studies.

Existing data can also be used to identify endophenotypes, such as existing gene-expression data, which are closer to the involved genes, but contribute to the etiology of diseases of interest [188]. Model organisms can also simplify the equation by allowing greater control over the environment [189]. A study in zebrafish used high-throughput methods to identify GxE related to exposure to abamectin [119]. Zebrafish allow for the use of high-throughput environmental methods with complex, vertebrate organisms [184]. Leveraging this system could even facilitate the high-throughput discovery of chemicals that exhibit genetically rooted differences in individual susceptibility across a population without any complex sequencing or statistical analysis. Using well-established, family-based experimental designs to assay chemicals and estimate important parameters like heritability could screen out chemicals that are unlikely to exhibit GxE [190,191]. Estimating heritability across the population of adverse effects using a well-established method could efficiently identify chemicals for further analysis, using the methods discussed in this paper, in the daunting chemical landscape that exists today. Combining well-established genetics approaches, endophenotypes, biological data, model organisms, high-throughput approaches, and a powerful analysis method such as the 2-step approach could create a compelling method to identify GxE related to endocrine and, particularly, thyroid disruption by punching through the complex pathways involved. Table 1 compares the approaches discussed here. Adding in functional validation to confirm results could be an efficacious approach to uncovering the etiology of thyroid diseases and the factors that contribute to susceptibility.

## 5. Conclusions

Current data on endocrine disruptors and gene-environment interactions are progressing rapidly on their own. Many sources point to genetic influences on the susceptibility of a population to the effects of these exposures, yet few studies have gone beyond the discovery of differential effects of EDCs on genetically diverse populations. This research is especially lacking in relation to thyroid disruptors. The groundwork exists to rapidly discover GxE in the context of EDCs. High throughput techniques to find signals of GxE in model organisms followed with high-dimensional genome-wide investigations could rapidly advance our understanding of GxE in this context. A new understanding of differential individual susceptibility to endocrine-disrupting chemicals across populations will help to develop more effective regulatory and mechanistic understandings that go beyond the current paradigm of EDC research to improve prevention and treatment of the adverse effects of exposure.

## Figures and Tables

**Table 1:** Comparison between methods that are described in the text that can be used to identify GxE. Sub-approaches are presented with over-arching approach that they draw from. Methods are presented with use case situations where they are generally considered useful.

Approach	Sub-Approaches	Use Cases	Citation
FAMILY-BASED	generalized estimating equations	Pedigree data is available and exposure mis-specification is a concern	Basson et al. (2016) [161], Sitlani et al. (2016) [162]
	heirarchical linear model	Pedigree data is available and type I error is a concern	
	linear mixed effects model	Pedigree data is available and type I error is a concern	
CASE-CONTROL	Penalized method with least absolute deviation loss function	When large genome-wide data is available and hierarchical “main effects, interactions” structure is a concern	Wu et al. (2018) [165]
	Similarity-based regression	When large genome-wide data is available and rare-variants with binary phenotypes are being investigated	Zhao et al. (2015) [124]
	linear mixed model	When large genome-wide data is available and multiple exposure are being investigated	BIOS Consortium (2016) [166]
	Parametric bootstrap	Removes need for permutation tests when large genome-wide data is available	Gauderman et al. (2017) [159], Coombes et al. (2018) [167]
CASE-ONLY	Traditional	Increases precision when independence between exposure and genetics can be assumed	Piegorsch et al. (1994) [169]
	Multiple maximum-likelihood	Increases precision and relaxes independence assumption	Umbach and Weinberg (1997) [170], Chatterjee and Carrol (2005) [173], Mukherjee and Chatterjee (2008) [171]
	Bayesian		
2-STEP	Likelihood ratio to traditional	Increases power and reduces multiple testing correction in situations where traditional case-control or case-case only approaches would be appropriate	Murcray et al. (2008) [171], Pare (2010) [174], Kooperburg and LeBlanc (2008) [175]
	Levene’s test to traditional		
	Marginal effects to traditional		
	Modified Pare et al.	Robust in situations with with multiple exposure and reduce type I error versus other 2-step approaches	Zhang et al. (2016) [160]
	Combined Pare and Kooperburg		
GENE-SET ANALYSIS (GSA)	Traditional	Increases power versus more traditional approaches	Biernacka et al. (2012) [176]
	With similarity regression	GSA when there are multiple covariates and opposite effects that may cancel each other out are a concern	Tzeng et al. (2013) [180]
	GESAT	Established method for user friendly GSA	Lin et al. (2013) [180]
META-ANALYSIS	NA	Situations where investigators want to combine data from multiple studies to identify possible gene-environment interactions	Shi et al. (2017) [168]

## Data Availability

Data used in the analysis in this paper are available in the supplementary material of Truong et al. (2020) [118].
